# Estado nutricional de pacientes pediátricos con deficiencia predominantemente de anticuerpos

**DOI:** 10.7705/biomedica.7398

**Published:** 2024-12-23

**Authors:** Lina M. Castaño-Jaramillo, Olga Rodríguez, Natalia Vélez-Tirado

**Affiliations:** 1 Servicio de Inmunología Clínica y Alergia Pediátrica, HOMI Fundación Hospital Pediátrico de La Misericordia, Bogotá, D. C., Colombia HOMI Fundación Hospital Pediátrico de La Misericordia HOMI Fundación Hospital Pediátrico de La Misericordia Bogotá, D. C. Colombia; 2 Servicio de Pediatría, HOMI Fundación Hospital Pediátrico de La Misericordia, Bogotá, D. C., Colombia HOMI Fundación Hospital Pediátrico de La Misericordia HOMI Fundación Hospital Pediátrico de La Misericordia Bogotá, D. C. Colombia

**Keywords:** estado nutricional, pediatría, enfermedades de inmunodeficiencia primaria, inmunodeficiencia común variable, agammaglobulinemia, disgammaglobulinemia, desnutrición, obesidad infantil., Nutritional status, pediatrics, primary immunodeficiency diseases, common variable immunodeficiency, agammaglobulinemia, dysgammaglobulinemia, malnutrition, pediatric obesity.

## Abstract

**Introducción.:**

La deficiencia predominantemente de anticuerpos es el grupo de errores inmunólogicos innatos más frecuente, sin embargo, hay poca información sobre el estado nutricional de los pacientes afectados.

**Objetivo.:**

Caracterizar el estado nutricional de pacientes colombianos con deficiencias predominantemente de anticuerpos.

**Materiales y métodos.:**

Se analizaron las historias clínicas de los pacientes con deficiencias predominantemente de anticuerpos en un hospital pediátrico de Bogotá.

**Resultados.:**

Se analizaron 55 historias clínicas. Los diagnósticos más frecuentes fueron la deficiencia específica de anticuerpos de polisacáridos, la deficiencia selectiva de inmunoglobulina A, la inmunodeficiencia común variable y la agammaglobulinemia.

Más del 70 % de los pacientes tenía infecciones sinopulmonares. La infección más frecuente fue la neumonía, seguida de la otitis media aguda y la sinusitis. El 45 % de los menores de cinco años tenía un peso adecuado para la talla, el 18 % tenía riesgo de desnutrición y el 18 % presentaba desnutrición aguda moderada; el 4,5 % sufría de obesidad, el 4,5 % tenía sobrepeso y el 9 % presentaba riesgo de sobrepeso.

En los mayores de cinco años, el 54 % tenía un índice de masa corporal adecuado, el 22,5 % tenía sobrepeso, el 9,6 % tenía riesgo de delgadez y el 9,6 % presentaba delgadez. Se encontró que el riesgo de talla baja y la talla baja fueron más frecuentes que la talla normal, y que los pacientes evaluados presentaron porcentajes de talla baja por encima de los reportados a nivel nacional.

**Conclusiones.:**

Debido a la epidemia de obesidad infantil, va a ser más frecuente encontrar sobrepeso u obesidad en niños mayores de cinco años, por lo que se considera que la talla baja puede ser un signo de alarma más sensible en pacientes con deficiencias predominantemente de anticuerpos.

## Abstract (en)

**Introduction.:**

Predominant antibody deficiency is the most frequent group of innate immunity errors, but information about patients’ nutritional status is scarce.

**Objective.:**

To characterize the nutritional status of Colombian patients with predominant antibody deficiencies.

**Material and methods.:**

We analyzed medical charts of patients with predominant antibody deficiency in a pediatric hospital in Bogotá.

**Results.:**

We analyzed 55 medical charts. The most frequent diagnoses were specific deficiencies of polysaccharide antibodies and immunoglobulin A, common variable immunodeficiency, and agammaglobulinemia. More than 70% of the patients had sinopulmonary infections, with pneumonia being the most frequent, followed by otitis and sinusitis. In children under five years, 45% had adequate weight for their height, 18% had a risk of malnutrition, and 18% had moderate acute malnutrition. Four-point-five percent had obesity, 4.5% showed overweight, and 9% had a risk of being overweight. Of those older than five years, 54% had an adequate body mass index, 22.5% showed overweight, 9.6% were at risk of thinness, and 9.6% were thin. We found that the risk of short stature and short stature perse were more frequent than the expected height in these patients. The percentages of patients with short stature were higher than those reported nationally.

**Conclusions.:**

Due to the epidemic of childhood obesity, it will be more frequent to find overweight or obesity in children above five years. Therefore, finding short stature could be a more sensitive alarm sign for predominant antibody deficiency.

Los errores inmunológicos innatos, también conocidos como inmunodeficiencias primarias, son un grupo de enfermedades con alteración en el desarrollo o en el funcionamiento del sistema inmunológico, de origen genético y con casi 50 genes asociados ^1^. El grupo de deficiencias predominantemente de anticuerpos es el más frecuente e incluye varias enfermedades como la agammaglobulinemia ligada al cromosoma X, la inmunodeficiencia común variable, la deficiencia selectiva de inmunoglobulina A y la deficiencia específica de anticuerpos polisacáridos con inmunoglobulinas y linfocitos B normales, entre otras ^2^.

Las deficiencias predominantemente de anticuerpos se caracterizan por la presencia de un defecto intrínseco del linfocito B que resulta en una alteración cuantitativa con linfopenia B, o en una cualitativa con disminución en la producción de inmunoglobulinas o inmunoglobulinas en un número normal, pero no funcionales ^3^. A pesar de ser el grupo de errores inmunológicos innatos que afecta un mayor número de pacientes, hay poca información sobre el estado nutricional de los afectados.

Históricamente, la desnutrición, la baja talla y el bajo peso se han considerado como signos de alerta para sospechar de forma temprana los errores inmunológicos innatos ^4^. Sin embargo, los pacientes con defectos humorales más leves pueden tener menor compromiso nutricional que aquellos con inmunodeficiencias combinadas ^5^^,^^6^.

En el presente estudio se buscó caracterizar el estado nutricional de pacientes colombianos con deficiencias predominantemente de anticuerpos, al momento del diagnóstico y en el seguimiento a los seis meses-.

## Materiales y métodos

Se llevó a cabo un estudio retrospectivo descriptivo, en el cual se analizaron los datos de los pacientes con diagnóstico de deficiencia predominantemente de anticuerpos de 2012 hasta 2022 en la Fundación Hospital Pediátrico de La Misericordia-HOMI en Bogotá.

Se incluyeron los pacientes con los siguientes diagnósticos: agammaglobulinemia, inmunodeficiencia común variable, síndrome hiper- IgM, deficiencia selectiva de isotipos (IgA, IgM), hipogammaglobulinemia transitoria de la infancia, deficiencia de subclases de IgG, deficiencia de cadenas ligeras, deficiencia específica de anticuerpos con inmunoglobulina y linfocitos B normales. Para los pacientes con deficiencia específicamente de anticuerpos con inmunoglobulina y linfocitos B normales, se implementaron los parámetros estadounidenses.

Se excluyeron los pacientes cuyos datos antropométricos no estaban disponibles.

Por protocolo institucional, los niños menores de dos años se pesaron en balanza para bebés y se midieron con el infantómetro; y los mayores de dos años, en balanza de pie y con tallímetro. Se registraron los datos respectivos en el *software* de historia clínica electrónica de la institución.

Las decisiones de practicar pruebas adicionales de laboratorio, hacer la valoración nutricional o hacer recomendaciones para un estilo de vida saludable las tomó el médico tratante según su criterio en cada consulta, ya que este estudio es retrospectivo y descriptivo, y no se contempló ninguna intervención en su metodología.

Se utilizaron los parámetros de la Resolución 2465 de 2016 del Ministerio de Salud y Protección Social de Colombia para determinar el riesgo de desnutrición, desnutrición moderada, desnutrición grave, sobrepeso, obesidad y talla baja.

Para la evaluación de la talla, se consideró talla adecuada aquellos con una talla para la edad mayor de -1 DE (desviación estándar); riesgo de talla baja, menor de -1 DE y mayor de -2 DE, y talla baja, en los casos con valores menores de -2 DE. Para los menores de 5 años, se evaluó el peso para la talla y se definió como adecuado con un valor de Z mayor de -1 DE y menor de +1 DE; riesgo de desnutrición menor, menor de -1 y mayor de -2 DE; desnutrición moderada menor de -2 y mayor de -3 DE, y desnutrición aguda grave en casos inferiores a -3 DE. Se definió riesgo de sobrepeso con un valor de Z mayor de +1 DE y menor de +2 DE; sobrepeso, mayor de +2 DE y menor de +3 DE, y obesidad, con más de +3 DE.

Para los menores de 5 años se evaluó el peso para la talla y se definió como adecuado con un valor de Z mayor de -1 DE y menor de +1 DE; riesgo de desnutrición, menor de -1 DE y mayor de -2 DE; desnutrición moderada, menor de -2 y mayor de -3 DE, y desnutrición aguda grave en casos menores de -3 DE.

Se definió riesgo de sobrepeso un valor de Z mayor de +1 y menor de +2 DE, sobrepeso mayor de +2 y menor de +3 DE y obesidad con valores mayor de +3 DE.

En mayores de 5 años se usó el IMC para la edad, definido como adecuado con un valor de mayor de -1 y menor de +1 DE; riesgo de delgadez, mayor de -1 y menor de -2 DE; delgadez, mayor de -2 DE; sobrepeso mayor de +1 y menor de +2 DE, y obesidad, valores mayores de +2 DE.

Se llevó a cabo un análisis descriptivo de la información. Las variables cuantitativas se analizaron mediante frecuencias, medidas de tendencia central y dispersión, según la distribución de los datos estimada con la prueba estadística de Shapiro-Wilk. Las variables cualitativas se representaron con frecuencias, porcentajes y proporciones. Para comparar las variables cuantitativas entre los grupos, se usó la prueba t de Student o, en el caso de datos no paramétricos, la prueba de Mann-Whitney, según los resultados obtenidos. Las variables cualitativas se compararon mediante la prueba de ji al cuadrado (c^2^) o la prueba exacta de Fisher. Para todas las pruebas se consideró estadísticamente significativo un valor menor de 0,05.

El análisis estadístico se realizó con el software SPSS™, versión 26 (SPSS Inc., Chicago, Illinois, USA). La información se presenta en tablas y gráficas de acuerdo con las características de los resultados.

### Aspectos éticos

Este proyecto fue aprobado por el Comité de Ética de la Investigación de la Fundación Hospital Pediátrico La Misericordia - HOMI, mediante el acta de aprobación No. 73, 569-23.

## Resultados

Se recolectaron datos de historias clínicas de 55 pacientes, 35 de sexo masculino (64 %). La mayoría de los pacientes (84 %) tenía, al menos, una comorbilidad. Las comorbilidades más frecuentes fueron el asma (44 %) y la rinitis (42 %).

**Figura 1.**
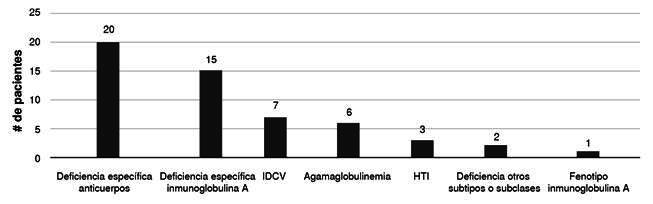
Diagnósticos específicos de pacientes con deficiencias predominantemente de anticuerpos.

Hubo cinco pacientes con síndromes genéticos definidos, dos con trisomía 21, uno con síndrome de Baraitser-Winter, uno con fiebre mediterránea familiar y uno con fibrodisplasia osificante progresiva. Cuatro pacientes tenían antecedentes de enfermedad autoinmunitaria, dos con artritis idiopática juvenil y dos con púrpura trombocitopénica inmunitaria, pero ninguno estaba recibiendo corticoesteroides al momento del estudio. Tres pacientes tenían antecedentes de neoplasia (leucemia o linfoma) y, tres pacientes, discinesia ciliar primaria. Los diagnósticos más frecuentes de las deficiencias humorales se pueden apreciar en la figura 1.

Más del 70 % tenía antecedentes de infecciones sinopulmonares; la más frecuente fue la neumonía en el 55 % de los pacientes, seguida de la otitis media aguda en el 31 % y de la sinusitis en el 9 %. En un quinto de los pacientes, coexistían infecciones sinusales y pulmonares altas o bajas.

El dato del número de infecciones sinopulmonares estaba disponible en 35 pacientes, con una mediana de cuatro episodios de neumonía (IQR = 15) y una mediana de otitis media aguda de cinco episodios (IQR = 7).

El 22 % de los pacientes tenía diarrea crónica, el 13 % había presentado infección urinaria y el 4 % había tenido abscesos cutáneos; el 7 % había padecido miocarditis y otras infecciones profundas. El 35 % de los pacientes recibía suplemento con inmunoglobulina a una dosis media de 627 mg/kg/ dosis (DE = 192). Ninguno de los pacientes con deficiencia específica de IgA estaba recibiendo inmunoglobulina sustitutiva, mientras que el 50 % de los pacientes con deficiencia específica de anticuerpos con inmunoglobulina y linfocitos B normales recibía suplencia.

**Figura 2.**
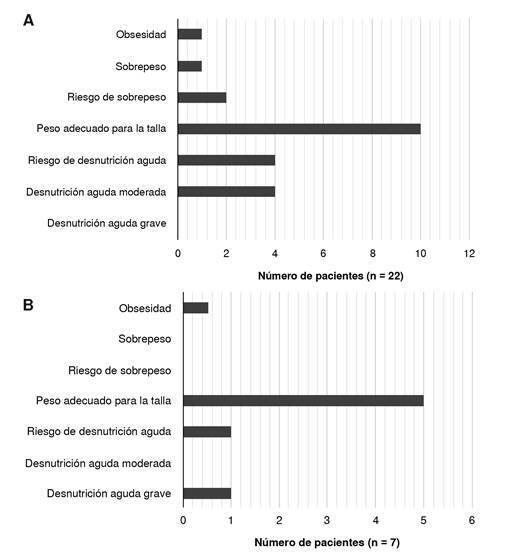
Peso para la talla en pacientes menores de cinco años con deficiencias predominantemente de anticuerpos. A: al momento del diagnóstico (n=22); B: durante el seguimiento (n=7)

En los menores de cinco años con errores inmunológicos innatos por deficiencia de anticuerpos, el 45 % tenía peso adecuado para la talla, el 4,5 % presentaba obesidad, el 4,5 % tenía sobrepeso y el 9 % estaba en riesgo de sobrepeso. Ningún niño cumplió con los criterios de desnutrición aguda grave, el 18 % estaba en riesgo de desnutrición y el 18 % en desnutrición aguda-moderada (figura 2, A y B).

**Figura 3.**
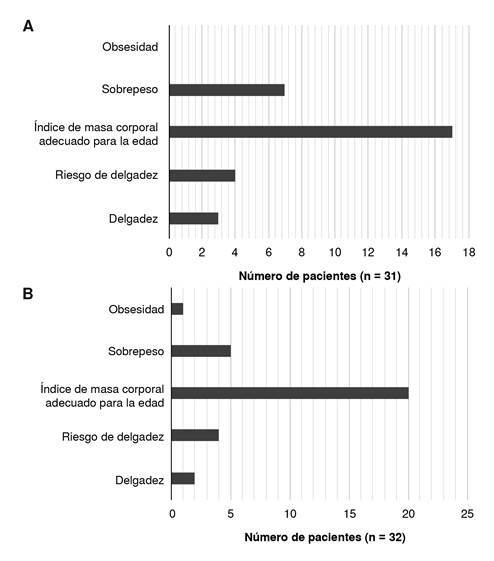
índice de masa corporal según la edad en pacientes mayores de cinco años con deficiencias predominantemente de anticuerpos. A: al momento del diagnóstico (n = 31); B: durante el seguimiento (n = 32).

En los mayores de cinco años, al momento del diagnóstico, el 54 % tenía un IMC normal para la edad, el 22,5 % sufría sobrepeso, el 9,6 % estaba en riesgo de delgadez y el 9,6 % estaban delgados. A los seis meses de seguimiento, se obtuvieron datos de solo siete pacientes por pérdida del seguimiento en la institución. Disminuyó el número de pacientes con alteraciones nutricionales, tanto de sobrepeso como de desnutrición. El número de pacientes con IMC normal para la edad aumentó al 64 %, pero, de nuevo, hubo pérdida de seguimiento (figura 3, A y B).

**Figura 4.**
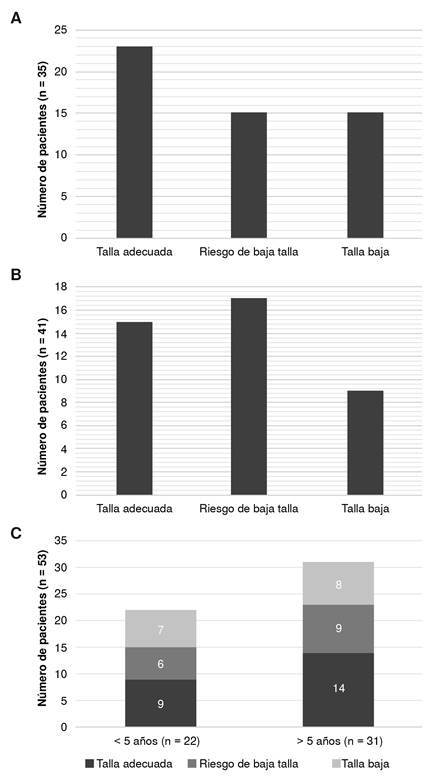
Talla para la edad de pacientes con deficiencias predominantemente de anticuerpos. A: al momento del diagnóstico (n = 53); B: durante el seguimiento (n = 41); C: al momento del diagnóstico diferenciado entre pacientes menores (izquierda ) y mayores (derecha) de cinco años.

En todos los pacientes a nivel global, se encontró talla baja en el 28 % y riesgo de talla baja en el 28 %, con una tendencia a la recuperación de la talla en el seguimiento dada por disminución de la proporción de niños con talla baja de todas las edades (figura 4, A, B y C). Incluso, al excluir los cinco pacientes con síndromes genéticos, de los cuales cuatro tenían talla baja y uno riesgo de talla baja, se encontró talla baja en el 23 % de los pacientes y riesgo de talla baja en el 30 %.

En los pacientes con deficiencia específica de anticuerpos con inmunoglobulina y linfocitos B normales, el 20 % tenía desnutrición o delgadez, el 20 % tenía riesgo de talla baja y el 15 % tenía talla baja. Solo un paciente con deficiencia específica de anticuerpos con inmunoglobulina y linfocitos B normales, tenía sobrepeso (5 %). Ninguno de los pacientes con deficiencia específica de IgA presentaba desnutrición, el 7 % tenía riesgo de talla baja y el 20 % tenía talla baja; el 20 % tenía sobrepeso. En los pacientes con agammaglobulinemia o inmunodeficiencia común variable, no se encontró ningún caso de desnutrición.

En este estudio no se encontraron diferencias significativas en el estado nutricional de los pacientes según el diagnóstico específico, la cantidad de infecciones sinopulmonares, la presencia de diarrea o la necesidad de terapia sustitutiva con inmunoglobulina. El estado nutricional de los pacientes al momento del diagnóstico y en el seguimiento a los seis meses, se muestra en las figuras 2 a 4.

## Discusión

A nivel mundial, las causas más frecuentes de inmunodeficiencia humoral son inmunodeficiencia común variable, agammaglobulinemia, hipogammaglobulinemia, deficiencia específica de anticuerpos con inmunoglobulina y linfocitos B normales y deficiencia específica de IgA ^7^^,^^8^. La inmunodeficiencia común variable es el error innato inmunológico sintomático más frecuente en la población adulta, pero en la población evaluada -exclusivamente pediátrica- representa la tercera causa más frecuente de deficiencias predominantemente de anticuerpos, después de la deficiencia específica de anticuerpos con inmunoglobulina y linfocitos B normales y deficiencia específica de IgA. Para la población estudiada, fue más frecuente la presencia de sobrepeso u obesidad que la desnutrición. Más de la mitad de los pacientes presentaron riesgo de talla baja o talla baja.

El estado nutricional de los pacientes pediátricos depende de varios factores, como el peso al nacer, el nivel socioeconómico y educativo, los hábitos de alimentación, la actividad física y la ingestión calórica ^9^. La relación entre el estado nutricional y el sistema inmunológico se ha estudiado previamente. Se ha documentado que la desnutrición crónica puede generar un estado de inmunodeficiencia secundaria con alteración de los mecanismos de inmunidad innata y adquirida, y mayor propensión a infecciones ^10^.

El retraso en el aumento de peso y en el crecimiento físico (fallo de medro) son reconocidos como signos de alarma para sospechar errores inmunológicos innatos ^11^. En 1959, Scrimshaw describió cómo la desnutrición aumenta el riesgo de infecciones recurrentes, graves y mortales, y cómo estas infecciones, a su vez, comprometían aún más el estado nutricional de los pacientes ^12^. Años después, la Fundación Jeffrey Modell incluyó el fallo del medro como signo de alerta para sospechar errores inmunológicos innatos ^13^.

En una cohorte del norte de Inglaterra, Subbarayan *et al.* evaluaron la utilidad de los signos de alarma para sospechar inmunodeficiencias primarias y encontraron que el uso de antibióticos intravenosos, el fallo de medro y la historia familiar de inmunodeficiencia, lograron identificar el 96 % de los pacientes con déficit de fagocitos y el 89 % de aquellos con defectos en los linfocitos T; el único signo de alarma que fue útil en el diagnóstico de las deficiencias predominantemente de anticuerpos fue la historia familiar ^14^.

El fallo de medro en pacientes con errores inmunológicos innatos es multifactorial; puede deberse a aumento de la demanda metabólica o a procesos infecciosos, estados inflamatorios persistentes, o a disminución de la ingestión y reducción de la absorción de nutrientes por inflamación o infección del tubo digestivo ^6^^,^^15^^,^^16^. El estado nutricional en estos pacientes es un factor determinante de la calidad de vida y la supervivencia. Incluso, en pacientes que son sometidos a trasplante de progenitores hematopoyéticos, el bajo peso para la edad se ha relacionado con la aparición de la enfermedad injerto contra huésped ^17^.

En años recientes, ha habido un aumento en la tasa de obesidad infantil a nivel mundial, derivado del gran consumo de alimentos procesados, ricos en carbohidratos y grasas, así como de un estilo de vida más sedentario ^18^^-^^20^. La obesidad no solo acarrea un riesgo metabólico y cardiovascular, sino que también, se ha asociado con alteraciones inmunológicas, como disminución en el número y la función de las células *natural killer*, menor eficacia de las vacunas, alteración en la proliferación de linfocitos o desregulación inmunológica asociada con adipocinas proinflamatorias ^21^^-^^24^. Sin embargo, hay poca información sobre la prevalencia de la obesidad en personas con errores inmunológicos innatos y su impacto en la función inmunológica. Es posible que un estado nutricional normal, e incluso de sobrepeso u obesidad, pueda estar asociado con un subdiagnóstico de las deficiencias predominantemente de anticuerpos.

Este es el primer estudio en que se evalúa el estado nutricional de pacientes con deficiencia predominantemente de anticuerpos en Colombia. En la cohorte evaluada, predominó el sexo masculino, como se ha descrito en otras cohortes ^15^^,^^25^. Las causas más frecuentes de deficiencias predominantemente de anticuerpos fueron la deficiencia específica de anticuerpos con inmunoglobulina y linfocitos B normales y la deficiencia específica de IgA, enfermedades que no tienen un comportamiento tan grave como el de las deficiencias de anticuerpos más importantes, como la agammaglobulinemia ligada al cromosoma X o la inmunodeficiencia común variable. A pesar de recibir inmunoglobulina sustitutiva, los pacientes con agammaglobulinemia ligada al cromosoma X mantienen bajos valores de IgA secretada y son propensos a infecciones e inflamación del tubo gastrointestinal ^26^.

En un estudio iraní, se identificó desnutrición en el 21 % de los pacientes y un riesgo o talla baja en más del 60 % ^27^. En la cohorte de este estudio, el 56 % de los pacientes presentaba riesgo de talla baja o talla baja, incluso al excluir los pacientes con síndromes genéticos diagnosticados. Se identificó talla baja en el 23 % de los pacientes, un valor dos o hasta tres veces mayor que la frecuencia de talla baja reportada en niños colombianos (11 % en menores de cinco años; 7,4 % en niños de 5 a 12 años y 9,7 % en adolescentes de 13 a 17 años) ^28^. En Turquía, Karhan *et al*. reportaron hasta el 50 % de desnutrición en pacientes con agammaglobulinemia ligada al cromosoma X. En general, en los pacientes con deficiencias predominantemente de anticuerpos se identificó un IMC menor de -2 DE en el 18 % de los pacientes, bajo peso para la edad en el 32 % y baja talla para la edad en el 18 % ^6^.

En la cohorte de USIDNET, con más de 1.400 pacientes con errores innatos de la inmunidad en los Estados Unidos, se encontró que el bajo peso sí es más frecuente en estos pacientes comparados con la población general. Sin embargo, es de resaltar que la frecuencia de sobrepeso (13,2 %) u obesidad (13,1 %) fue cuatro veces mayor que la de bajo peso (6,6 %) en la población pediátrica ^15^. En un estudio multicéntrico en pacientes con agammaglobulinemia en Italia, también se encontró que el sobrepeso (26 %) y la obesidad (12 %) son más frecuentes que el bajo peso (5 %) ^29^.

En el presente estudio, en los menores de cinco años, fue más frecuente el riesgo de desnutrición o la desnutrición aguda, que el riesgo de sobrepeso, sobrepeso u obesidad. Sin embargo, en los mayores de cinco años, el sobrepeso fue más frecuente que el riesgo de delgadez o la delgadez.

Los datos recolectados no permiten establecer una asociación, pero posiblemente este fenómeno tiene relación con una mayor frecuencia de infecciones -especialmente gastrointestinales- con mayor efecto nutricional en menores de cinco años. La frecuencia de sobrepeso estuvo acorde con lo observado previamente en la población colombiana ^28^. Además, se encontró que era más frecuente que los pacientes cursaran con riesgo de talla baja o talla baja, que con una talla adecuada para su edad. Aunque es escaso el número de pacientes con datos sobre seguimiento, la tendencia observada es la recuperación de la talla y la disminución de la relación entre peso y talla. Por el carácter retrospectivo de este trabajo, no es posible determinar otras intervenciones que pudieron haber generado esta tendencia, más allá del manejo de los errores inmunológicos innatos.

El asma fue la comorbilidad más frecuente en los pacientes con deficiencia primaria de anticuerpos. Weinberger *et al*. encontraron una prevalencia de asma en el 33 % de aquellos con inmunodeficiencia común variable y en el 10 % de aquellos con agammaglobulinemia, similar a lo descrito en el presente trabajo ^30^. Muchos pacientes con deficiencias predominantemente de anticuerpos tienen historia clínica sugestiva de enfermedad alérgica de la vía aérea (asma); sin embargo, durante su abordaje se encontraron niveles normales de IgE. Además, no es posible objetivar la sensibilización a aeroalérgenos.

En la experiencia de este estudio, algunos pacientes clasificados como asma de difícil manejo con exacerbaciones infecciosas frecuentes cursaban con deficiencia específica de anticuerpos con inmunoglobulina y linfocitos B normales, con mejoría de sus manifestaciones pulmonares al iniciar tratamiento con inmunoglobulina sustitutiva.

La definición de deficiencia específica de anticuerpos con inmunoglobulina y linfocitos B normales incluye una historia de susceptibilidad aumentada a infecciones sinopulmonares, niveles cuantitativamente normales de inmunoglobulinas y una respuesta ineficiente a las vacunas polisacáridas.

Es una de las deficiencias predominantemente de anticuerpos más subdiagnosticadas, ya que requiere un alto índice de sospecha y pruebas muy especializadas para su diagnóstico ^31^.

Actualmente, hay muy poca información sobre el estado nutricional de pacientes con errores inmunológicos innatos, especialmente en Latinoamérica, por lo que es difícil comparar los resultados de esta serie de casos con otras poblaciones.

Las principales limitaciones de este trabajo son que es un reporte retrospectivo de un solo centro y el pequeño tamaño de la muestra. Los autores consideran que, a futuro, es importante la colaboración con otros centros especializados en Colombia, dado el número restringido de pacientes. La proporción de pacientes reportados de Latinoamérica en la base de datos de la *Latin American Society for Immunodeficiencies* (LASID), muestra una proporción de diagnósticos de inmunodeficiencias humorales similar a la descrita en este trabajo ^32^. Dado que el HOMI es un centro de referencia, esta serie parece ser representativa de la situación real del país y de Latinoamérica.

Aunque el bajo peso y la falla de medro deben hacer sospechar errores inmunológicos innatos, ya que estos son más frecuentes en estas condiciones patológicas que en la población general, no se puede ignorar que, ante la epidemia de obesidad infantil, aumenta la posibilidad de encontrar pacientes con dichos errores y sobrepeso u obesidad, especialmente en los mayores de cinco años. Un estado nutricional adecuado, el sobrepeso o la obesidad, no descartan que el paciente curse con deficiencias predominantemente de anticuerpos. Sin embargo, más de la mitad de los pacientes pueden presentar talla baja, un signo de alarma más sensible para sospechar deficiencias predominantemente de anticuerpos que el bajo peso para la talla.
